# Research on Assimilation of Unmanned Aerial Vehicle Remote Sensing Data and AquaCrop Model

**DOI:** 10.3390/s24103255

**Published:** 2024-05-20

**Authors:** Wei Li, Manpeng Li, Muhammad Awais, Leilei Ji, Haoming Li, Rui Song, Muhammad Jehanzeb Masud Cheema, Ramesh Agarwal

**Affiliations:** 1 National Research Center of Pumps, Jiangsu University, Zhenjiang 212013, China; lmp13011586313@163.com (M.L.); awais@ujs.edu.cn (M.A.); leileiji@ujs.edu.cn (L.J.); lhm17826076819@163.com (H.L.); srui0227@163.com (R.S.); 2 Institute of Fluid Engineering Equipment Technology, Jiangsu University, Zhenjiang 212009, China; 3 Wenling Fluid Machinery Technology Institute, Jiangsu University, Wenling 317525, China; 4 Faculty of Agricultural Engineering and Technology, PMAS-Arid Agriculture University, Rawalpindi 46000, Pakistan; 5 Louis McKelvey School of Engineering, Washington University, St. Louis, MO 63114, USA

**Keywords:** UAV remote sensing, canopy coverage, AquaCrop crop model, parameter assimilation

## Abstract

Taking the AquaCrop crop model as the research object, considering the complexity and uncertainty of the crop growth process, the crop model can only achieve more accurate simulation on a single point scale. In order to improve the application scale of the crop model, this study inverted the canopy coverage of a tea garden based on UAV multispectral technology, adopted the particle swarm optimization algorithm to assimilate the canopy coverage and crop model, constructed the AquaCrop-PSO assimilation model, and compared the canopy coverage and yield simulation results with the localized model simulation results. It is found that there is a significant regression relationship between all vegetation indices and canopy coverage. Among the single vegetation index regression models, the logarithmic model constructed by OSAVI has the highest inversion accuracy, with an R^2^ of 0.855 and RMSE of 5.75. The tea yield was simulated by the AquaCrop-PSO model and the measured values of R^2^ and RMSE were 0.927 and 0.12, respectively. The canopy coverage R^2^ of each simulated growth period basically exceeded 0.9, and the accuracy of the simulation results was improved by about 19.8% compared with that of the localized model. The results show that the accuracy of crop model simulation can be improved effectively by retrieving crop parameters and assimilating crop models through UAV remote sensing.

## 1. Introduction

In recent years, with the biochemical research of crop growth, the precision and operability of crop models have become higher and higher [1]. However, due to the complexity and uncertainty of the crop growth process, the model can only achieve a relatively accurate simulation on a single point scale. With the development of remote sensing technology, the efficiency of obtaining large-area field information has been improved, and the combination of remote sensing technology and crop models has a good application prospect [2]. Currently, the commonly used data assimilation algorithms can be divided into three types: updating method, forcing method, and optimization method.

The update method iteratively changes the initial values and parameters of the crop model by minimizing the cost function of the difference between simulated values and remote sensing inversion values, so as to make the simulation closer to the inversion values [3]. This calibration method can effectively improve the simulation accuracy of yield, but it is difficult to apply on a large scale due to the huge number of calculations. The update method is efficient and easy to implement, but it also has some disadvantages. First, the measurement data required for each process usually require interpolation, especially when integrating optical remote sensing data, and complex clouds can greatly reduce the number of available observations. Second, it forcibly breaks the simulation process because it replaces the intermediate result with an external input. Finally, it is easy to transfer error to the model without considering the measurement error. Because of these shortcomings, some recent studies have considered the issue of forced data assimilation. Awais et al. [4] compared the forcing method with the updating method and found that when the observed values were assimilation in weekly steps, the updating method could improve the error by 65%, while the forcing method could only achieve 20%. However, from the perspective of technology and operation, it was still difficult to have available LAI observation results for the whole growing season [5].

In order to improve the accuracy of model simulation, crop parameters obtained from remote sensing inversion are forced to replace the original parameter results of the model, and crop growth simulation is continued [6]. Assimilation is carried out during the simulation, intervening only when observational data are available. Compared to the update method, the forced method can show good performance even with small and infrequent data observations and reduce processing time. In addition, the method can solve the uncertainty problem of simulated and assimilated data. However, this requires modifications to the models themselves, and not all models allow for this intervention. The most commonly used update technologies include the Kalman filter, particle filter, and ensemble Kalman filter. Soenen et al. [7] reduced the crop model results to a single field, assimilated LAI time series into the crop model simulation using an integrated Kalman filter, and simulated and verified the field corn yield in several states in the United States, with an error of only about 10%.

The optimization method adjusts the initial data of the crop model according to the crop parameters of remote sensing inversion, obtains the optimized simulation value, constructs the cost function for it, and makes it converge continuously with the remote sensing inversion parameters until it is the smallest. Commonly used optimization algorithms include the PSO algorithm [8], SA algorithm [9], SZE-UA algorithm, etc. [10]. These optimization algorithms have reliable global optimization capabilities and flexibility of input and objective functions. Particle swarm optimization has various applications in remote sensing, and it is often used in remote sensing image processing [11,12,13] but also in agriculture, where the most common application is the optimal calibration of crop models, such as the WOFOST model [14], SAFY model [15], DSSAT model, or AquaCrop model [16,17,18,19]. Guo et al. [20] used this algorithm to combine the PROSAIL canopy reflectance model with the WheatGrow crop model based on the vegetation index. Betbeder et al. [21] estimated soybean yield and leaf area index by constructing an assimilation model with the SAFY-WB model combined with the NDVI index, and the accuracy was 0.86. Liu Feng et al. [22,23,24,25] constructed an assimilation system using the hybrid evolution algorithm, the extremely fast simulated annealing algorithm, and the CERES-Wheat model, and they conducted simulation tests on the leaf area index of wheat. From the results, the application of the assimilation system constructed on wheat was relatively reliable.

With the continuous improvement in the accuracy requirements of crop models, the current assimilation model still remains in the simulation of field crops and single growth periods, and the application scope and accuracy of assimilation models need to be improved urgently. Therefore, it is necessary to build a new assimilation model to break the barrier of the application scope of crop models and improve the accuracy of crop models.

## 2. Materials and Methods

### 2.1. Field Experiment Data

#### 2.1.1. Overview of the Test Area

**Figure 1:**
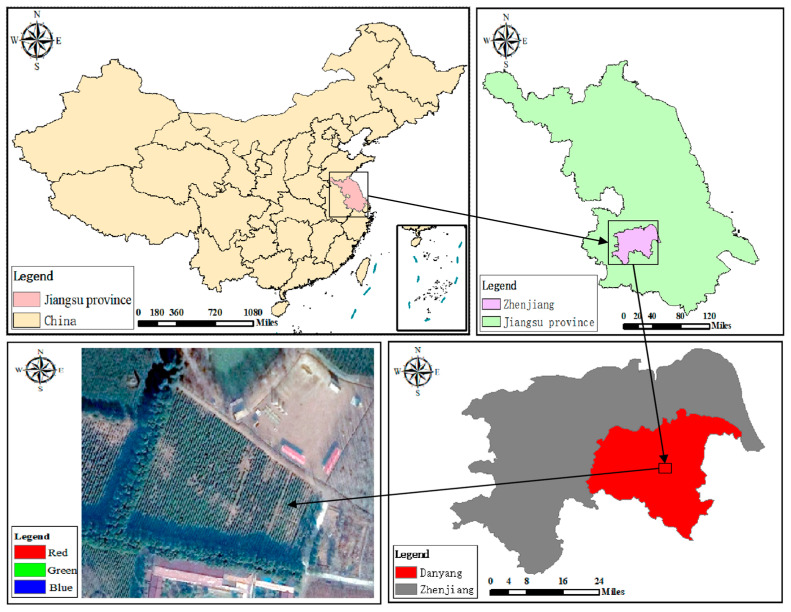
Overview of the test area.

The experimental data were collected in a tea field (32°1′00″ N, 119°4’00″ E) in Danyang City, Zhenjiang City, Jiangsu Province [26]. The tea variety planted in the field is MAO Green Tea, and the experimental site is shown in Figure 1. The average annual temperature ranges from 13.6 °C to 16.1 °C. The average temperature in winter is 3.0 °C. The extreme minimum temperature usually occurs in January or February in winter, the average temperature in summer is 25.9 °C, and the extreme maximum temperature usually occurs in July or August in midsummer. The seasonal distribution characteristics of annual precipitation are obvious, in which the summer precipitation is concentrated, accounting for almost half of the annual precipitation, the winter precipitation is the least, accounting for about one-tenth of the annual precipitation, and the spring and autumn precipitation account for about 20% of the annual precipitation.

The tea tree variety planted in the test area is Longjing 43. The canopy height is from 0.7 m to 0.8 m, and the canopy width is from 0.8 m to 0.9 m. The seeding method is single row loading, with the spacing of large rows being 150 cm and the spacing of plants being 30 cm. The harvesting method is one season a year, the time is mid-March every year, and cutting correction is carried out after harvest. Because the tea trees in the test area include many old tea trees, lifting treatment is required in May every year. The tea farm is relatively large and flat, providing a suitable site for remote sensing measurement by drones. During the experiment, the overall environment was good, the weather was clear, and the average wind speed was less than 1.5 m/s.

#### 2.1.2. Field UAV Remote Sensing Data Acquisition

**Figure 2:**
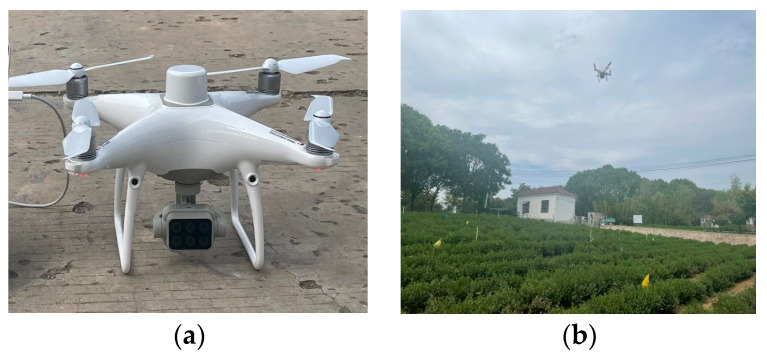
(**a**) DJI multi-rotor UAV. (**b**) Remote sensing image field acquisition.

The UAV remote sensing system adopts the DJI Phantom 4 RTK designed by DJI. Its specific parameters are shown in Table 1. The platform is equipped with 1 RGB sensor and 5 monochrome sensors for multispectral imaging, as shown in Figure 2a. The UAV flight experiment was completed from early May 2022 to mid-November 2023, with flight times from 7:00 to 17:00, as shown in Figure 2b. UAV flights were operated by DJI Ground Station Professional software (v4.0.10), and the DJI A3 flight control was used.

Multispectral sensors have bandwidths of about 10–15 nm in the electromagnetic spectrum region of blue, green, red, red-edged, and near infrared. The geographic resolution of the multispectral sensor has strong universality for agricultural applications, and the spectral response is positive at the canopy level. To this end, multispectral sensors are often used in the agricultural sector at a fairly low cost. The multispectral sensor detects the light absorbed by plants in four distinct parts: green and red light and two infrared bands invisible to the human eye. Table 2 shows some necessary specifications for multispectral sensors. After tea was picked and pruned in the whole test area, a multispectral camera equipped with a DJI Spirit 4 UAV platform was applied for image acquisition. As shown in Table 2, the multispectral camera equipped with 6 lenses can simultaneously acquire one RGB image and five band images, including red, green, blue, red-edge, and NIR.

### 2.2. Remote Sensing Image Processing

**Figure 3:**
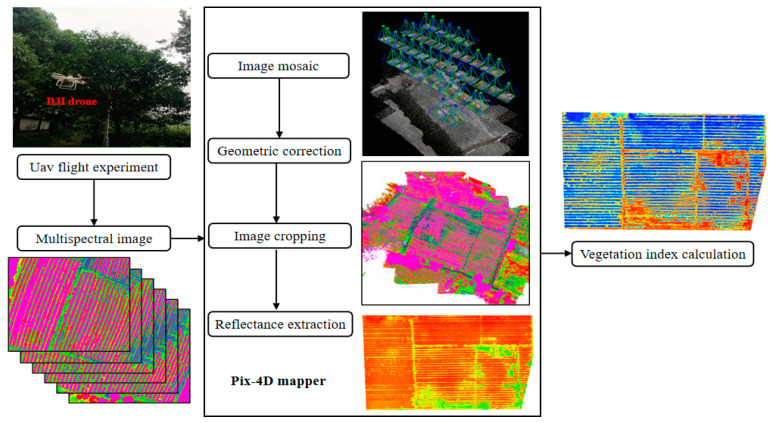
Remote sensing data processing flowchart.

**Figure 4:**
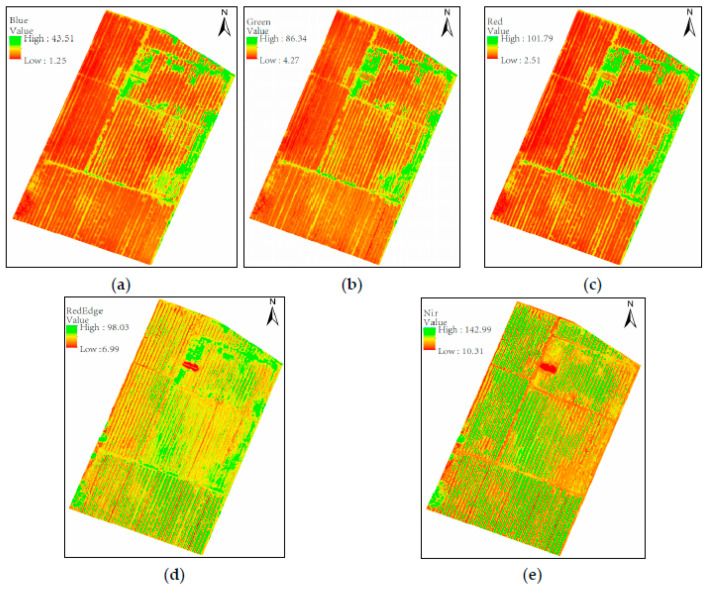
Reflectivity RdYlGn diagram of various wavebands: (**a**) blue, (**b**) green, (**c**) red, (**d**) red edge, (**e**) NIR.

When the remote sensing image is acquired by a UAV, the multispectral image has a certain distortion and deviation due to the atmospheric non-uniformity and the sensor itself. Therefore, when applying a multispectral image, it must be preprocessed first [27,28,29,30]. The main method is to use image control points and image processing algorithms to correct the image. In this paper, the pre-processing of multispectral images is introduced in detail. The main process is shown in Figure 3, including image stitching, radiation correction, geometric correction, and image clipping. The single image obtained by a UAV flight cannot fully reflect the whole picture of the test area. In this paper, Pix4D-Mapper(4.0.24) softwareis used to splice remote sensing images. In order to correct the deformation caused by remote sensing image splicing, geometric correction must be carried out on the spliced images. The specific process of correction is performed to obtain the coordinates of the image control points through RTK and then pair the coordinates with the image control points in the Mosaic image, so as to realize the geometric correction of the Mosaic image. Each image size has different degrees of deviation, and the image also contains some information of non-studied areas. Therefore, before the spectral reflectance analysis, the image must be properly cut to ensure that the size of the image is equal in different spectral ranges, so as to facilitate spectral estimation and finally obtain a relatively complete multispectral reflection image, as shown in Figure 4.

### 2.3. Canopy Coverage Extraction

**Figure 5:**
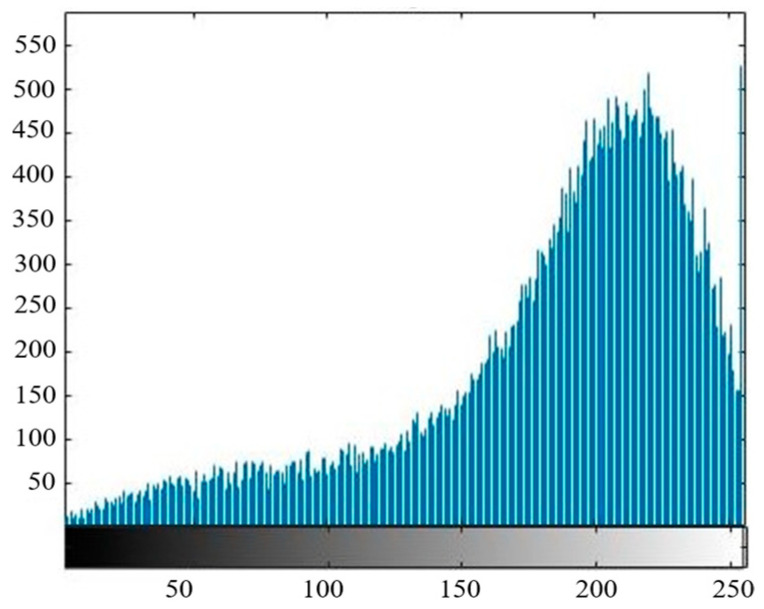
Histogram of gray frequency distribution of red-band image.

**Figure 6:**
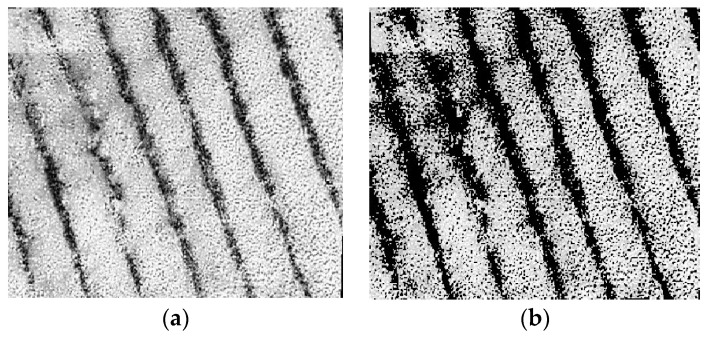
Soil pixel removal results: (**a**) original grayscale map, (**b**) soil background elimination.

In this paper, the ratio of the color pixels of the canopy to the total pixels of the photo is extracted to obtain the canopy coverage. Considering that the sensitivity of the tea canopy to different bands is slightly different, the red band with the strongest sensitivity of the tea canopy is adopted to extract the canopy coverage. At the same time, in order to improve the accuracy of calculation, this paper uses the binary Otsu algorithm to eliminate the soil background. The main process of the algorithm is as follows: (1) Read the red-band image and convert it into a grayscale image to obtain the grayscale matrix of the image; (2) obtain the histogram of the gray frequency distribution of the red-band image and set the optimal gray threshold, as shown in Figure 5; (3) compare the size between the gray value of each pixel and the threshold value; (4) if the gray value is lower than the threshold, the output gray value is 0, otherwise the original gray value is retained; and (5) output the gray-scale image after background region segmentation, as shown in Figure 6. After removing the soil background, the canopy coverage can be obtained by calculating the proportion of canopy pixels in the total pixels through Matlab.

### 2.4. Calculation of Vegetation Index

The vegetation index (VI) is calculated by combining multi-band spectral information and can be used to measure crop growth [31,32,33]. Through band calculation, the influence of air reflection on the multispectral sensor in different time periods is reduced, the acquisition speed is fast, and the acquisition range is wide. At present, the vegetation index is widely used in the monitoring of vegetation phenotype data, crop growth and development status, and ecological environment status. The traditional extraction method of the vegetation index is based on the reflectance of a certain band in the pixel spectral curve, but in practical applications, a single band will make some specific spectral features (such as green, red, etc.) submerged or appear with a “false green” and “false red” phenomenon [34,35,36,37]. Therefore, it is of great significance to analyze and extract spectral features from different angles and levels. For example, the vegetation index calculated according to the red, blue, and near-infrared bands can effectively monitor the growth state of the vegetation canopy. In essence, the vegetation index comprehensively considers the reflection and absorption characteristics of healthy green plants in different bands and performs a certain mathematical transformation of multi-band reflectance to enhance crop vegetation information. In this paper, 10 kinds of commonly used vegetation indices are calculated according to the reflectance of the tea canopy, which is extracted and eliminates the influence of crop shadows, as shown in Table 3.

### 2.5. Basic Principles of AquaCrop Model

AquaCrop is a hydrological model developed by the Food and Agriculture Organization of the United Nations (FAO) to assess the water use efficiency and yield response of crops. The model aims to help farmers, agricultural planners, and policymakers better understand and manage agricultural water resources. AquaCrop models can predict crop growth, yield, and water use efficiency based on different soil, crop, and climate conditions. Its expression is as follows [49]:

(1) $$\left(\frac{Y-Y_{m}}{Y}\right)=K_{y}\left(\frac{ET-ET_{0}}{ET}\right)$$

where *Y* is the potential crop yield, kg/m^2^; *Y*_m_ is the actual crop yield, kg/m^2^; *K*_y_ is the scaling factor of crop yield response to water; *ET* is the potential crop evapotranspiration, mm; and *ET*_0_ is the actual crop transpiration, mm.

The AquaCrop model can accurately simulate the soil water situation of the crop growth period and divide the soil depth *d* and the crop growth time *t* into several segments; the size of each segment is ∆d and ∆t, assuming that the soil depth on the node *t_j_* at a certain time is *d_i_*; and the soil water balance (*d_i_*,*t_j_*) of the node (*ρ_i_*_,_*_j_*,m^3^·m^−3^) is calculated as follows:

(2) $$\rho_{i,j}=\rho_{i,j-1}+\Delta \rho_{i,\Delta t}$$

In the formula, *ρ_i_*_,*j*−1_ is the soil water content (m^3^·m^−3^) of the time node *t_j_*_−1_ on the node (di, tj), ∆*ρ_i_*, and ∆t is the soil water change corresponding to the unit time node ∆t.

(3) $$\Delta \rho_{i,\Delta t}=\Delta \mathrm{Wrs}+\Delta \mathrm{Wleak}+\Delta \mathrm{Welsr}+\Delta \mathrm{Esoil}+\Delta \mathrm{Ecrop}$$

where ∆Wrs is the water redistributed to soil layer *i*; ∆Wleak is water that seeps into soil layer *i*; ∆Welsr is water that seeps into soil layer *i* in addition to surface runoff; ∆Esoil is the evapotranspiration of the soil layer *i*; and ∆Ecrop is the water consumed by evapotranspiration crops in soil layer *i*.

### 2.6. Particle Swarm Optimization Assimilation Principle

Particle swarm optimization (PSO) is a random optimization algorithm proposed by Wolfram in the 1980s. It adopts the idea of particle swarm and transfers the global optimal solution to the local optimal solution through certain rules to realize the optimization of the global optimal solution. The application steps of PSO are as follows [49]:

(1) Firstly, the position and velocity of each particle are determined by random distribution, and then the relevant information (such as velocity and position) is taken as a special weight in the current solution space at each random position;
(2) A better (or worse) solution in the space of a set of possible solutions found is selected, which is the location of the optimal value of the current state (objective function);
(3) Finally, the particle swarm is moved to a new position according to certain rules;
(4) After the new position is generated, the influence of each particle on the position of the minimum value of the objective function in the current state (that is, the weight) is calculated, so as to achieve the purpose of optimizing the objective function.

Because the algorithm treats each particle as a living, intelligent agent, any member of the group can have an impact on other members, so it also has individual intelligence; When new information enters the group, it will make decisions together with all members of the group and optimize the objective function. In this way, the whole group can continuously search for the global and local optimal solutions in the algorithm.

(4) $$pbest(i,t)=\underset{\begin{array}{c} i=1,\ldots ,N\\ k=1,\ldots ,M \end{array}}{\arg\min}\left[f\left(x_{i}^{t}\right)\right]$$

(5) $$gbest(t)=\underset{\begin{array}{c} i=1,\ldots ,N\\ k=1,\ldots ,M \end{array}}{\arg\min}\left[f\left(x_{i}^{t}\right)\right]$$

where *pbest* is the optimal position through which the particle passes; *gbest* is the optimal location of the particle swarm; *i* is the particle index; *N* is the particle swarm size; *t* is the number of iterations; *f* is fitness function; and *P* is where the particle is. The particle velocity and position update formulae are as follows:

(6) $$v_{id}^{t+1}=\omega v_{id}^{t}+c_{1}r_{1}\left(p_{id,pbest}^{t}-x_{id}^{t}\right)+c_{2}r_{2}\left(p_{d,gbest}^{t}-x_{id}^{t}\right)$$

(7) $$x_{i}^{t+1}=x_{i}^{t}+v_{i}^{t+1}$$

where $v_{id}^{t}$ is the *d* dimensional velocity vector of particle *i* in the *t* iteration; $x_{id}^{t}$ is the *d* dimensional position vector of particle *i* in the *t* iteration; *ω* is the inertia weight; *r_1_* and *r_2_* are uniformly distributed random variables in the interval [0, 1]; *c*_1_ is the individual learning factor; *c*_2_ is the group learning factor; $p_{id,pbest}^{t}$ is the historical optimal position of particle *i* in the *d* dimension in the *t* iteration; and $p_{d,gbest}^{t}$ is the historical optimal position of the group in the *d* dimension in the *t* iteration.

### 2.7. Model Evaluation Index

The evaluation of model performance mainly includes two aspects: goodness of fit and prediction accuracy. In this paper, determination coefficient R^2^ and root mean square error RMSE are used to evaluate the model comprehensively.

(1) Coefficient of determination R^2^

The determination coefficient R^2^ is the fitting result between the simulated value and the actual value, and its value ranges from 0 to 1. The closer R^2^ is to 1, the better the fitting degree of the regression equation and the better the prediction result of the target variable. The closer R^2^ is to 0, the less confident the model’s predictions are. The specific expression is as follows:

(8) $$R^{2}=\frac{\sum_{i=1}^{n}{\left(M_{i}-\bar{M}\right)}^{2}}{\sum_{i=1}^{n}{\left(S_{i}-\bar{M}\right)}^{2}}$$

where *n* is the total number of samples; *M_i_* is the measured value; *S_i_* is the simulation value; and $\bar{M}$ is the mean of the predicted value.

(2) Root Mean Square Error RMSE

The root mean square error (RMSE) measures the deviation between the simulated value and the actual value and is sensitive to the outliers in the data. The smaller the RMSE, the higher the accuracy of the model and the better the inversion effect. The larger the RMSE, the lower the accuracy of the model. The specific expression is as follows:

(9) $$RMSE=\sqrt{\sum_{i=1}^{n}\frac{{\left(S_{i}-M_{i}\right)}^{2}}{n}}$$

## 3. Results and Analysis

### 3.1. Correlation Analysis of Canopy Coverage

Before modeling the vegetation index and canopy coverage, it is necessary to study the correlation between the vegetation index and canopy coverage. The purpose is to preliminarily screen 10 planting cover indices and use the vegetation index with a strong correlation for modeling. This paper uses the Pearson correlation coefficient to measure the correlation between the vegetation index and canopy coverage. The Pearson correlation coefficient is defined as the quotient of covariance and standard deviation between two variables. The specific expression is shown as follows:

(10) $$r_{X,Y}=\frac{\mathrm{cov}(X,Y)}{s_{X}s_{Y}}=\frac{E[(X-m_{X})(Y-m_{Y})]}{s_{X}s_{Y}}$$

**Figure 7:**
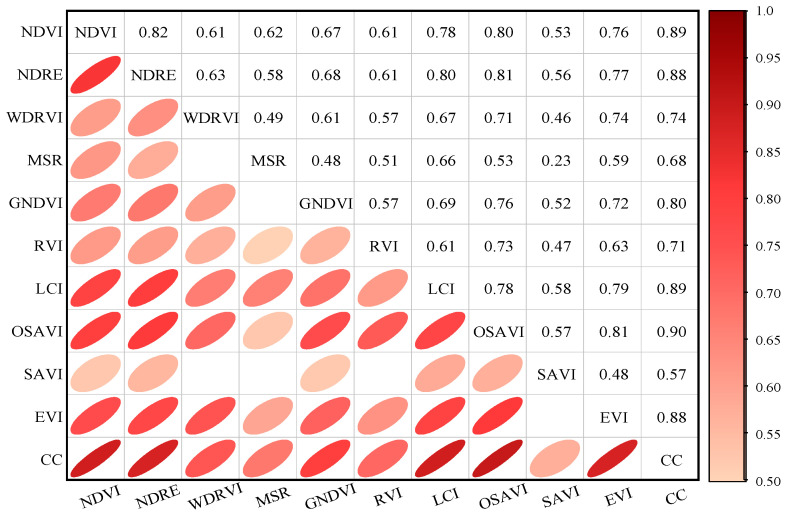
Matrix plot of vegetation index and Pearson correlation coefficient of canopy coverage.

**Figure 8:**
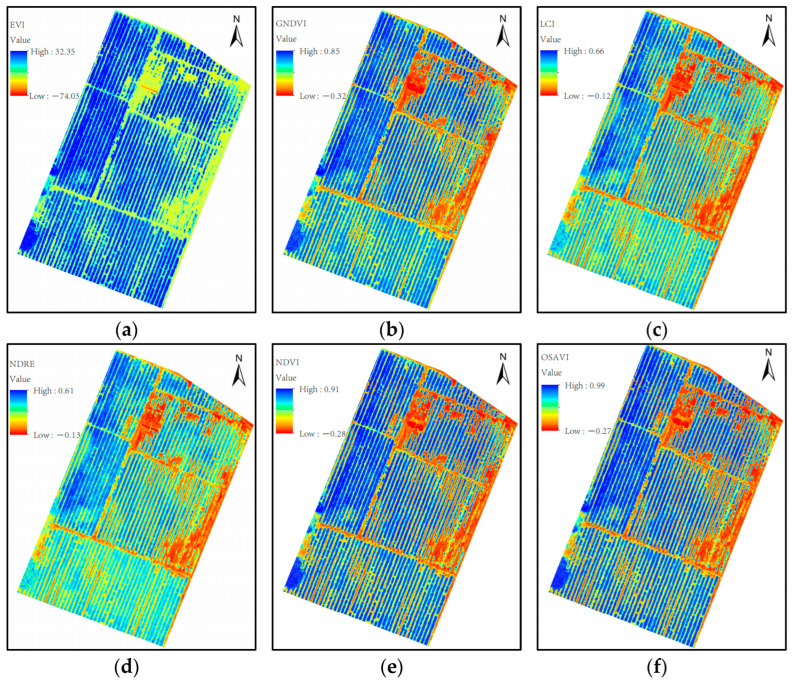
Schematic diagram of vegetation index in the experimental area: (**a**) EVI, (**b**) GNDVI, (**c**) LCI, (**d**) NDRE, (**e**) NDVI, (**f**) OSAVI.

Figure 7 shows the Pearson correlation coefficient matrix of the canopy coverage and vegetation index. The value represents the Pearson correlation coefficient among variables. The closer the absolute value is to 1, the stronger the correlation between variables is. The white box in the figure indicates that the correlation coefficient is lower than 0.5. As can be seen from Figure 7, the correlation coefficients between different vegetation indices and canopy coverage are concentrated in the range of 0.57 to 0.9, in which NDVI, NDRE, GNDVI, LCI, OSAVI, and EVI are highly correlated with canopy coverage, with correlation coefficients ranging from 0.8 to 0.9. WDRVI, MSR, and RVI were strongly correlated with canopy coverage, with correlation coefficients ranging from 0.6 to 0.8 (0.74, 0.68, and 0.71, respectively). The correlation between SAVI and canopy coverage was the worst, and the correlation coefficient was only 0.57. Therefore, in this study, NDVI, NDRE, GNDVI, LCI, OSAVI, and EVI were selected to establish inversion models with canopy coverage, so as to select the optimal canopy coverage inversion model. The calculation results of six vegetation indices selected in the test area are shown in Figure 8.

### 3.2. Canopy Coverage Inversion Based on Vegetation Index

#### 3.2.1. Modeling Results of Canopy Coverage Inversion Model

**Figure 9:**
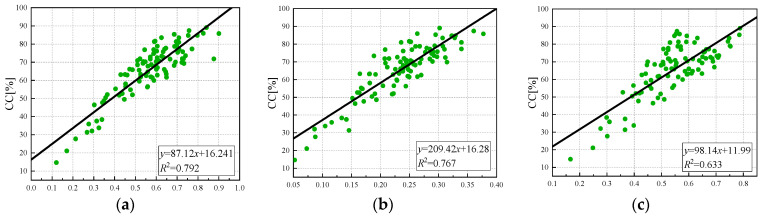

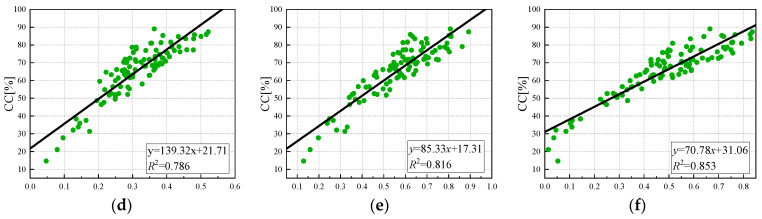
Univariate linear regression model of vegetation index and canopy coverage: (**a**) NDVI, (**b**) NDRE, (**c**) GNDVI (**d**) LCI, (**e**) OSAVI, (**f**) EVI.

The linear regression model is simple in structure and can effectively invert the canopy coverage. As shown in Figure 9, a unary linear regression model of the vegetation index and canopy coverage was established. The vegetation index that had the best correlation with canopy coverage was EVI (R^2^ = 0.85), and the worst correlation was GNDVI (R^2^ = 0.633). The coefficients of determination with NDVI, NDRE, LCI, and OSAVI were 0.79, 0.767, 0.786, and 0.816, respectively. The model accuracies are EVI, OSAVI, NDVI, LCI, NDRE, and GNDVI from high to low. It can be observed that at a high canopy coverage, the degree of dispersion is relatively large. Therefore, the nonlinear relationship between the vegetation index and canopy coverage can be established to obtain the best model to simulate canopy coverage.

**Figure 10:**
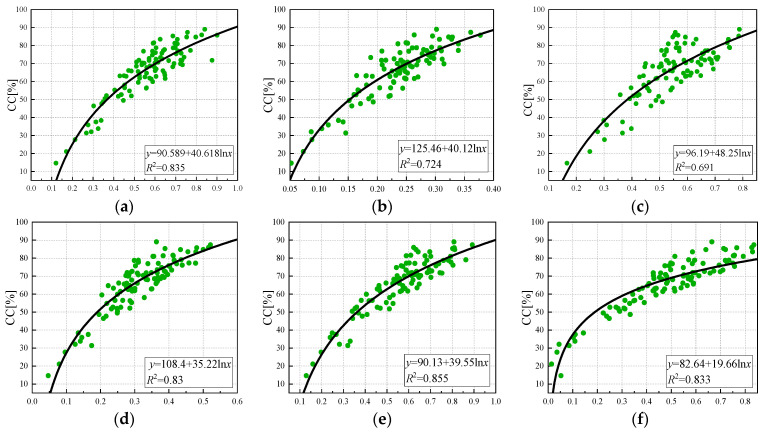
Logarithmic regression model of vegetation index and canopy coverage: (**a**) NDVI, (**b**) NDRE, (**c**) GNDVI (**d**) LCI, (**e**) OSAVI, (**f**) EVI.

As shown in Figure 10, a logarithmic model of the vegetation index and canopy coverage was constructed, and the overall accuracy was better than that of the linear regression model. The best and worst correlations between the vegetation index and canopy coverage were OSAVI (R^2^ = 0.855) and GNDVI (R^2^ = 0.691), respectively, and the determination coefficients of NDVI, NDRE, LCI, and EVI were 0.835, 0.724, 0.83, and 0.833, respectively. The accuracy of estimation was in the following order: OSAVI, NDVI, EVI, LCI, NDRE, and GNDVI. At the later stage of tea growth, the phenomenon of supersaturation is more likely to occur, so the canopy coverage will be underestimated at the later stage of growth. Therefore, the relationship between the vegetation index and canopy coverage may present an exponential model.

**Figure 11:**
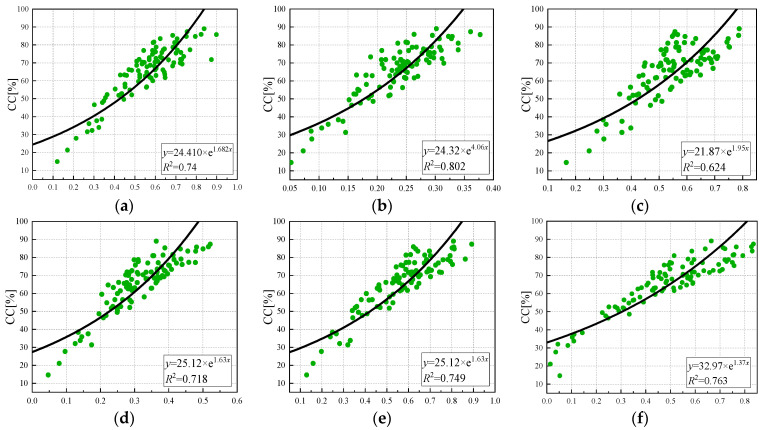
Regression model of vegetation index and canopy coverage index: (**a**) NDVI, (**b**) NDRE, (**c**) GNDVI (**d**) LCI, (**e**) OSAVI, (**f**) EVI.

As shown in Figure 11, the exponential model relationship between the vegetation index and canopy coverage was established. The inversion accuracy of the exponential regression model was lower than that of the logarithmic and linear regression models, especially when the canopy coverage was low, and the inversion accuracy was poor. The correlation between NDRE and the leaf area index was the best (R^2^ = 0.802), and GNDVI was the worst (R^2^ = 0.624). The coefficients of determination of NDVI, LCI, OSAVI, and EVI were 0.74, 0.718, 0.749, and 0.763. From high to low, the estimation accuracy was EVI, OSAVI, NDVI, NDRE, LCI, and GNDVI.

**Figure 12:**
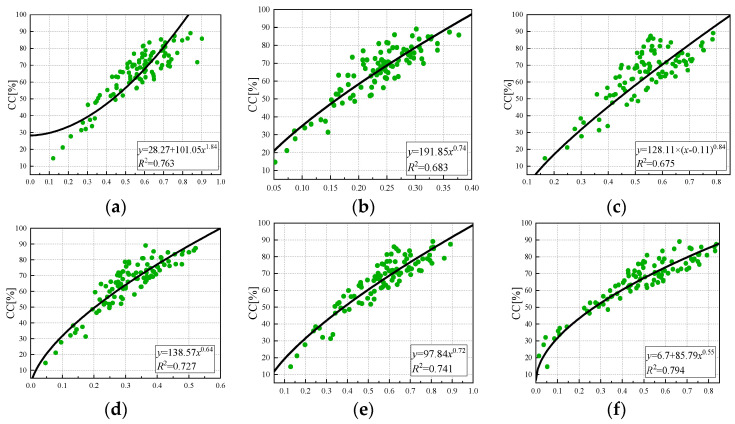
Power function regression model of vegetation index and canopy coverage: (**a**) NDVI, (**b**) NDRE, (**c**) GNDVI (**d**) LCI, (**e**) OSAVI, (**f**) EVI.

As shown in Figure 12, a power function model relationship between the vegetation index and canopy coverage was established, and there was a certain power function relationship between each vegetation index and canopy coverage. The correlation between vegetation index EVI and the leaf area index was the best (R^2^ = 0.794), and the coefficient of determination between vegetation index GNDVI and NDRE was the lowest (R^2^ = 0.68). The determination coefficients of NDVI, LCI, and OSAVI were 0.763, 0.727, and 0.741, respectively. The accuracy of estimation was EVI, NDVI, OSAVI, LCI, NDRE, and GNDVI in descending order.

**Figure 13:**
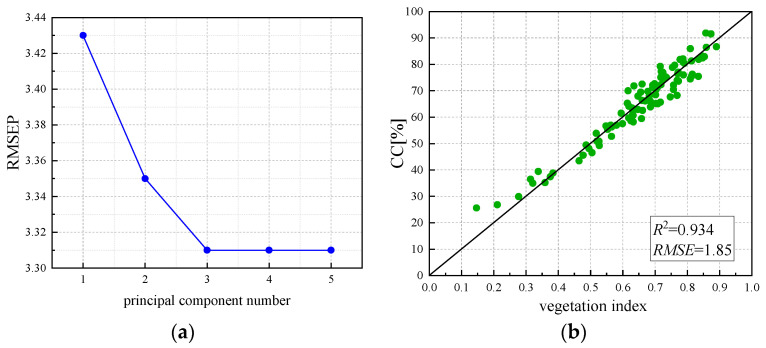
(**a**) RMSEP analysis; (**b**) PLSR modeling.

The above regression models are all regression models constructed with a single vegetation index. According to previous studies, the accuracy of the model constructed with a single component is often lower than that of the multi-component model. Therefore, based on the single index, in this study, five vegetation indices (NDVI, OSAVI, EVI, NDRE, and LCI) with high correlation were selected for partial least squares regression (PLSR) and the model was established [50,51,52]. By analyzing the prediction error root mean square (RMSEP), it is found that when the principal component number reaches 3, RMSEP is 3.31, that is, the principal component number RMSEP will continue to increase at this time and will not decrease, as shown in Figure 13a. Therefore, it is considered that when the principal component number is 3, the fitting effect of the established PLSR model can achieve the best result, and the required data are the least. Three vegetation indices, NDVI, OSAVI, and EVI, were selected to construct the PLSR regression model. The fitting results are shown in Figure 13b, and the R^2^ and RMSE are 0.934 and 1.85, respectively. Relatively high R^2^ and low RMSE indicate that the accuracy of the constructed PLSR model is higher than that of the regression model constructed by the single vegetation index, and the accuracy is the highest among all inversion models.

#### 3.2.2. Canopy Coverage Inversion Model Test

Through modeling studies, it was found that there was a strong correlation between most vegetation indices and canopy coverage. In order to further determine the correlation between the two, 20 groups of field experiment data were selected to verify the inversion accuracy of the established regression model. According to the analysis of the results of the above regression models, the regression model with the best fitting effect of each vegetation index was selected to invert the canopy coverage, as shown in Table 4. Since the correlation of the regression models established by GNDVI was below 0.7, it was only moderately correlated, so it was no longer considered in the model verification. Through the analysis of linear, logarithmic, exponential, power function, and PLSR regression models, the following was found: Among the regression models constructed by a single vegetation index, the logarithm regression models constructed by NDVI, NDRE, LCI, and OSAVI had the highest accuracy, and R^2^ and RMSE were 0.835, 0.802, 0.83, and 0.855 and 6.135, 6.714, 6.217, and 5.753, respectively. All of them were above 0.8. The optimal regression model constructed using EVI is the linear regression model with R^2^ and RMSE of 0.853 and 5.796, respectively, second only to the logarithmic regression model constructed by OSAVI. The accuracy of the PLSR regression model constructed by multiple vegetation indices is higher than that of a single vegetation index, and R^2^ and RMSE are 0.934 and 1.85, respectively.

**Figure 14:**
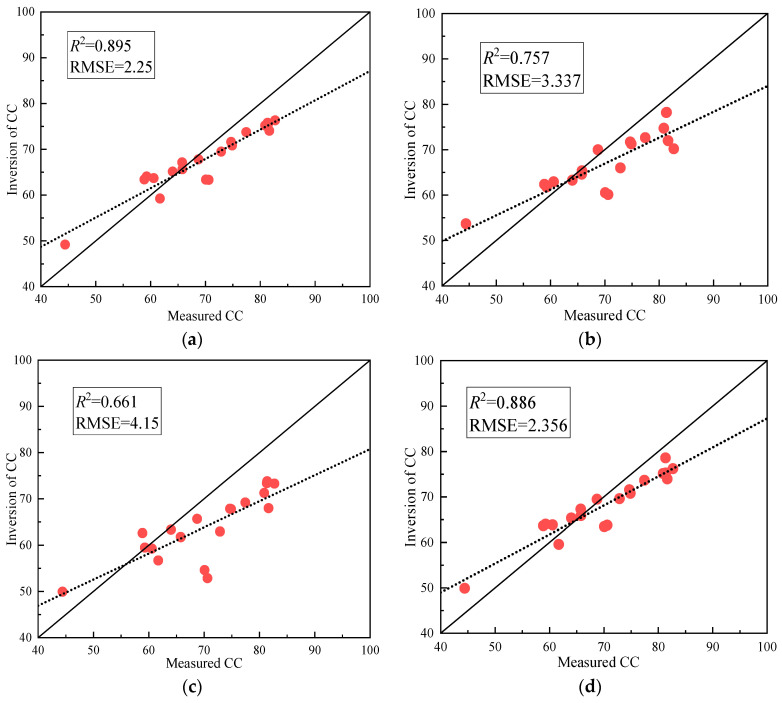

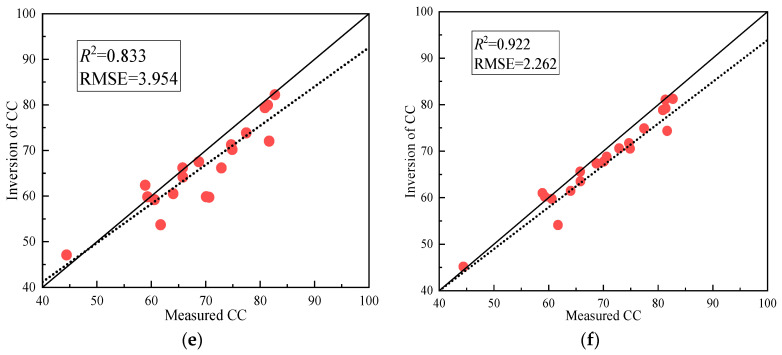
Validation of the optimal estimation model for canopy coverage: (**a**) NDVI, (**b**) NDRE, (**c**) LCI, (**d**) OSAVI, (**e**) EVI, (**f**) PLSR.

As can be seen from the model verification results in Figure 14, the estimation accuracy of the regression model constructed by NDRE and LCI decreased, and R^2^ and RMSE were 0.757 and 0.661 and 3.337 and 4.15, respectively. NDVI, OSAVI, EVI, and PLSR still maintained a good correlation, and R^2^ and RMSE were 0.895, 0.886, 0.833, and 0.922 and 2.253, 2.356, 3.954, and 2.262, respectively. The accuracy of the verified models from high to low is PLSR, NDVI, OSAVI, EVI, NDRE, and LCI. Therefore, the PLSR regression model was selected in this study for canopy coverage inversion.

### 3.3. Research on Aquacrop Model Assimilation Based on PSO

#### 3.3.1. PSO Assimilation Process

**Figure 15:**
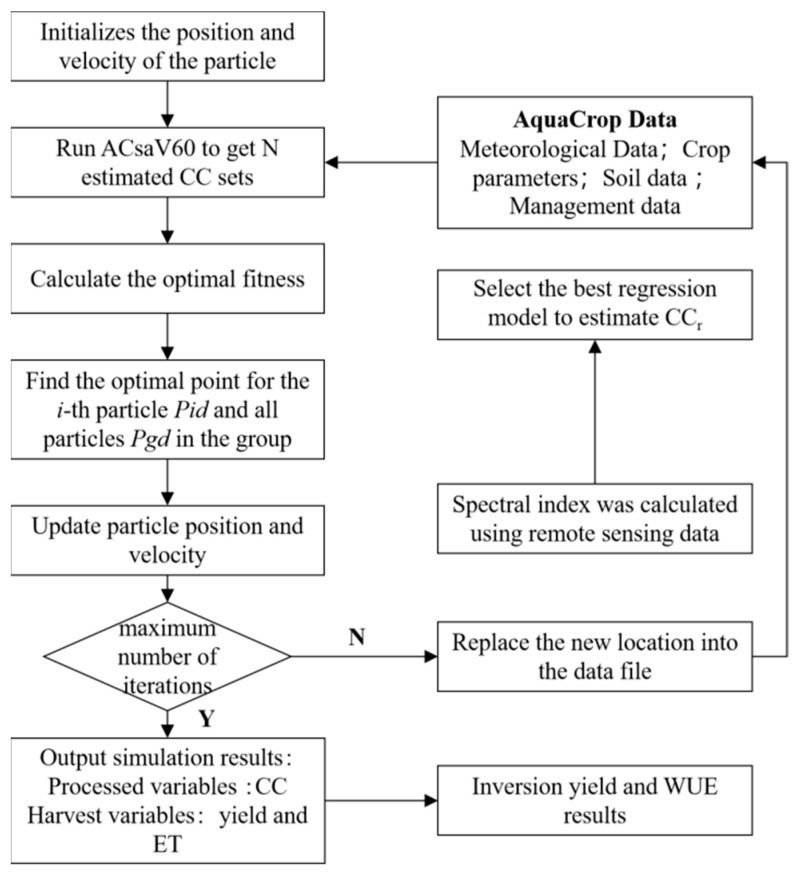
Data and AquaCrop model assimilation flowchart based on PSO method.

The assimilation process of remote sensing data and the AquaCrop model based on the PSO algorithm is described in detail in Figure 15. In this study, canopy coverage is taken as the intermediate assimilation dynamic variable. The specific steps are as follows:

(1) The initial value (position) and particle velocity are determined. The adjusted parameters include nine crop parameters, *CC_ini_*, *den*, *mcc*, *wp*, *hi*, *kcb*, *Tmg*, *Tupper,* and *Tbase*. The initial values and value ranges of the parameters are shown in Table 5.

(2) MATLAB was used to run the ACsaV60.exe plug-in, integrate with the required data, and output analog CC (CC_s_).
(3) The PLSR regression model was used to estimate canopy coverage CC (CC_r_).
(4) The cost function of CC_s_ for model simulation and CC_r_ for remote sensing inversion was constructed so that its value converges continuously until it reaches the minimum, at which time the optimization algorithm also finds the best input parameters. The cost function selected in this study is shown in Equation (11).

(11) $$f=\sqrt{\frac{1}{n}\sum \left(\frac{CC_{s}-CC_{r}}{CC_{s}}\right)}$$

In the formula, CC_s_ is the canopy coverage simulated by AquaCrop. CC_r_ is the canopy coverage of remote sensing inversion.

#### 3.3.2. Optimal Fitness Analysis of Particle Swarm

**Figure 16:**
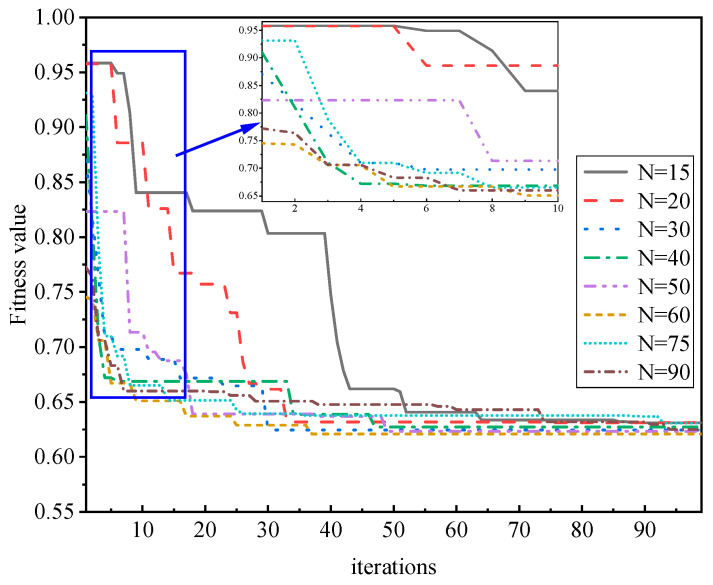
Particle swarm optimization fitness analysis.

Based on the AquaCrop model, this paper uses experimental data from 2022 to conduct optimization iterative evaluation and an analysis of the particle swarm optimization algorithm. The number of particles in the particle swarm and the number of iterations are compared and analyzed, and the analysis results are shown in Figure 16. The number of particles was set to 15, 20, 30, 40, 50, 60, 75, and 90 for 100 iteration optimization calculations. As shown in Figure 16, the particle optimization algorithm shows good performance in the AquaCrop model and remote sensing data assimilation process. With the increase in iterations, the optimal fitness of particles steadily declines and gradually tends to a stable state. This phenomenon indicates that the root mean square error (RMSE) between CC_s_ and CC_r_ gradually decreases and reaches the minimum value, and the whole regression process has become stable. Therefore, it can be considered that the particle swarm optimization algorithm has good feasibility for the assimilation process of the AquaCrop model and remote sensing data. After comparing the level of the particle number setting, it is found that when the number of particles is 60, with an increasing number of iterations, the optimal fitness reaches 0.744 after 20 iterations, and the optimal value remains stable and no longer changes at about 0.621 after 37 iterations. In this study, fitness values of 15, 20, 40, 50, 75, and 90 particles were found to be higher than those of 60 particles. In addition, regardless of the number of particles, the optimal fitness is very high in the initial iterations. When the number of particles is 15, the highest fitness value is 0.958, and when the number of particles is 60, the lowest fitness value is 0.744. With multiple iterations of the optimization algorithm, the optimal fitness value in the optimization process drops sharply in the first 20 iterations. However, after increasing the number of iterations, the optimal fitness value gradually approaches the global optimal solution. From the 30th iteration, the optimal fitness value basically presents a stable state. Although it is close to the optimal solution at this time, it is still not the best state. With further iterations, the optimal fitness value with a particle number of 60 reached a stable period at 37 iterations, and at about 50 iterations, all particle numbers entered a stable period and did not change significantly [53].

Based on the above analysis, this paper sets the number of particles as 60 and the number of optimization iterations as 40 as parameters for assimilating remote sensing images. These parameters are related to the assimilation process of remote sensing data and crop models at the spatial scale of the experimental area. By analyzing the optimal fitness value of the particle swarm optimization algorithm, the accuracy of the optimization search of the particle swarm optimization algorithm can be effectively evaluated, so that the CC_r_ can be coupled to the crop growth model and more accurate simulation results can be obtained.

#### 3.3.3. Estimation Accuracy Evaluation Based on Assimilation Model

**Figure 17:**
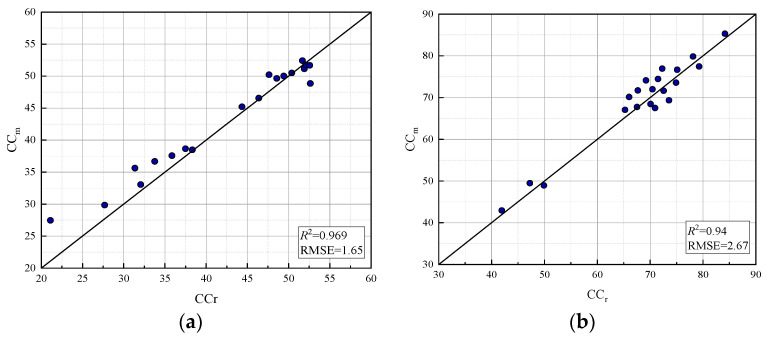

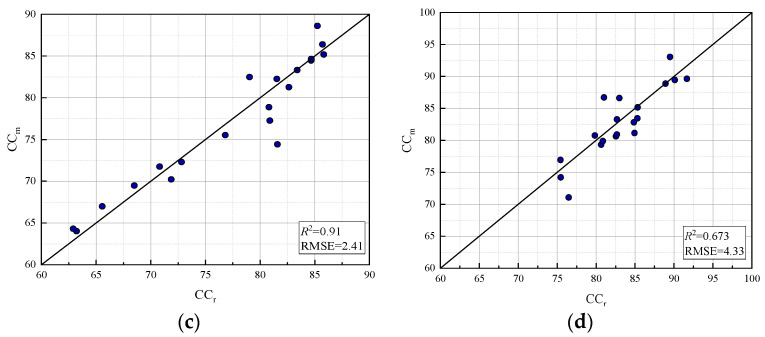
Relationship between assimilation and measured CC at each growth stage: (**a**) spring tea growing period, (**b**) summer tea growing period, (**c**) autumn tea growing period, (**d**) winter tea growing period.

In this study, canopy coverage was used as a dynamic variable to adjust the relevant parameters of the AquaCrop model, the assimilation between the AquaCrop model and remote sensing data was realized by particle swarm optimization (PSO), and the AquaCrop-PSO assimilation model was constructed. The PLSR regression model was used to invert CC_r_, and the RMS RMSE between CC_r_ and CC_s_ simulated by the assimilation model was calculated. As shown in Figure 17, the simulation accuracy of the spring, summer, autumn, and winter growth stages of crops was simulated and analyzed by the AquaCrop-PSO model. Remote sensing data were collected in April, July, October, and December. The accuracy of the spring, summer, and autumn growth periods is higher, and the R^2^ reaches above 0.9, among which the highest is 0.96 in spring, and the lowest is 0.67 in winter. The results showed that the AquaCrop-PSO assimilation model could be applied to the estimation of tea canopy coverage in the experimental area.

**Figure 18:**
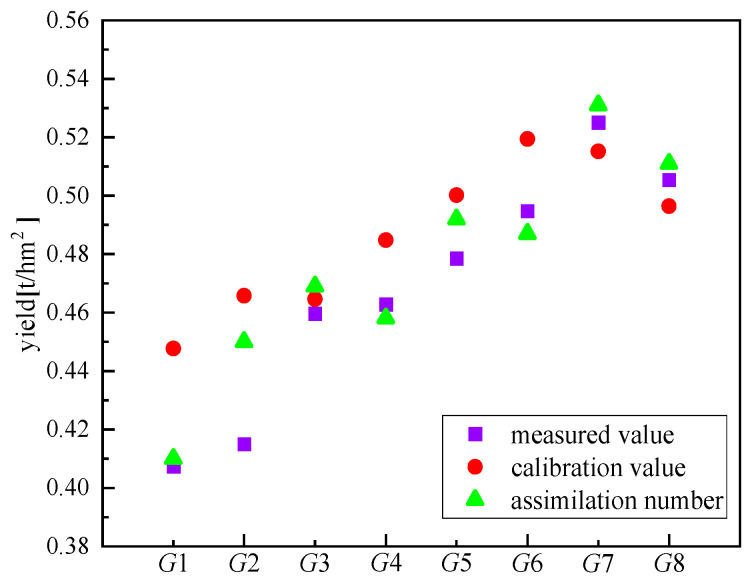
Comparison chart of yield prediction and measured value of calibration model and assimilation model.

In order to test the ability of the assimilated model in predicting yield, the yield predicted by the AquaCrop model, the yield predicted by the AquaCrop-PSO assimilation model, and the measured yield were selected for comparative analysis. The results are shown in Figure 18. The predicted results of the calibration and assimilation model are higher than the measured values, and the assimilated values are mostly in the middle of the calibrated values and the measured values. The results showed that the assimilated model corrected the high predicted value of the AquaCrop model. The R^2^ of the actual yield and the simulated yield after calibration was 0.727 and the RMSE was 0.24. The R^2^ of the actual yield and the simulated yield of the assimilated model was 0.925 and the RMSE was 0.12. Higher R^2^ and lower RMSE validate the simulation accuracy of the AquaCrop-PSO assimilation model on yield.

## 4. Conclusions

This paper analyzed the UAV flight test data, used the Otsu algorithm to eliminate the soil background in RGB images, extracted the canopy coverage of tea gardens, and selected the six planting indices with the highest correlation with the measured canopy coverage for regression analysis and verification, so as to build the optimal canopy coverage inversion model. Based on the obtained canopy coverage, the AquaCrop-PSO assimilation model was constructed through particle swarm optimization and the AquaCrop model assimilation experiment, and the comparison and verification were carried out. The following conclusions are drawn:

(1) Ten vegetation indices related to canopy coverage were selected, and their Pearson correlation coefficients were tested. Finally, six vegetation indices (NDVI, NDRE, GNDVI, LCI, OSAVI, and EVI) with correlations above 0.8 were selected to establish regression models with canopy coverage. The results showed that all vegetation indices had a significant regression relationship with canopy coverage. Except EVI, the logarithmic regression model had the best simulation effect, and the logarithmic regression model constructed by OSAVI had the highest estimation accuracy (R^2^ = 0.855).
(2) Multiple vegetation indices (NDVI, NDRE, LCI, OSAVI, and EVI) were selected to construct a partial least squares regression model. RMSEP analysis found that the simulation accuracy was optimal when the principal component was 3. Therefore, NDVI, OSAVI, and EVI were selected to establish a PLSR model. The simulated and verified R^2^ and RMSE reached 0.93 and 1.85 and 0.94 and 2.26, respectively, which are the optimal regression models and can be used to invert tea canopy coverage.
(3) AquaCrop-PSO was used to simulate the canopy coverage of tea in each growing period. The accuracy of spring, summer, and autumn was higher, and the R^2^ was above 0.9, while the accuracy of winter was lower, and the R^2^ was 0.67. In the simulation of production, R^2^ and RMSE simulated by AquaCrop-PSO were 0.927 and 0.12, which improved the simulation accuracy compared with the calibrated AquaCrop model.

## 5. Outlook

Due to the wide application of crop models in the world, the assimilation processing of crop models by obtaining crop parameters through remote sensing or sensors is an effective means to improve the accuracy of crop models, and many scholars have invested in relevant research. De et al. [54] used a low-resolution satellite microwave sensor (SWI) to retrieve crop LAI values and assimilated them through an integrated Kalman filter (EnKF) to correct the water balance error in the WOFOST crop model and evaluate crop yield. Due to the low accuracy of satellite inversion, although some progress has been made, the accuracy of the WOFOST model has not been effectively improved. Lu et al. [55] assimilated the bivariables of soil water and canopy coverage into the AquaCrop model, and the simulation accuracy of yield and soil water was greatly improved. The R2 of the assimilation model reached 0.695, but it was difficult to obtain parameters, and the two-parameter assimilation resulted in the overall robustness of the model being reduced. Guerif et al. [56] obtained assimilation coupling between crop LAI and a crop model by using a radiative transfer model, and they combined spectral reflectances into the TSAVI vegetation index to greatly improve LAI inversion accuracy. The relative error of yield estimation of the assimilation model proposed by Guerif ranged from 0.6% to 2.6%, effectively improving the accuracy of the crop model, but they did not consider crop seasonal changes. The data do not cover the whole growing period. In this study, the low-altitude remote sensing of an unmanned aerial vehicle was used to construct an accurate inversion model of crop coverage. The Aquacrop-PSO assimilation model was constructed through the particle swarm optimization algorithm and AquaCrop model assimilation experiment, and the assimilation accuracy was above 0.9 considering the changes in the whole growing period of crops. However, by comparing the previous research results, some shortcomings of this study are also found, and the following prospects are proposed:

(1) This study is based on the particle swarm optimization algorithm to conduct assimilation research on remote sensing data and crop models. Only one algorithm is selected to conduct assimilation research on remote sensing data and crop models. A variety of different assimilation algorithms should be selected for further analysis to enhance the applicability and expansibility of the assimilation model. Because the existing assimilation algorithms are slow and time-consuming, it is necessary to improve the assimilation efficiency if the assimilation calculation is to be carried out on a large regional scale.
(2) This study was carried out based on the climatic conditions in the eastern coastal area of China, which are rainy and humid, so the model needs to be further adjusted and applied under similar climatic conditions in other regions. Meanwhile, the application of the assimilation model under other arid climatic conditions needs to be tested and explored.
(3) The crop studied in this paper is tea. As a water-loving crop, the simulation accuracy of the growth model of tea may change when compared with that of other xerophytic crops. The next step is to design other crop experiments on this basis, collect the actual parameters of different crops, and compare and analyze the crop parameters under simulated and measured conditions.

## 6. Patents

(1) An intelligent decision system for farmland irrigation based on digital word generation (patent number: 202211508391.9).
(2) An intelligent farmland irrigation decision-making system based on the remote sensing data inversion of an unmanned aerial vehicle (patent number: 202110604577.3).

## Figures and Tables

**Table 1:** DJI multi-rotor UAV parameters.

Aircraft Parameters	Value
Takeoff weight	1487 g
Diagonal wheelbase (without paddle)	350 mm
Maximum flight altitude	6000 m
Maximum ascent speed	6 m/s (Automatic flight); 5 m/s (Manually operated vehicle)
Maximum descent speed	3 m/s
Maximum horizontal flight speed	50 km/h (positioning mode); 58 km/h (Attitude mode)
Time of flight	27 min
Operating frequency	5.725 GHz–5.850 GHz

**Table 2:** Multispectral sensor parameters.

Band	Wave Length (nm)	Bandwidth (nm)	Pixel Size
Red	650	40	1.2
Green	560	40	1.2
Blue	450	40	1.2
RedEdge	730	10	1.2
NIR	840	40	1.2
RGB	-	-	16

**Table 3:** Formulae for calculating the vegetation indices.

Vegetation Index	Formula
NDVI (normalized differential vegetation index) [38]	$\mathrm{NDVI}=\frac{\mathrm{NIR}-\mathrm{Red}}{\mathrm{NIR}+\mathrm{Red}}$
NDRE (normalized difference red edge index) [39]	$\mathrm{NDRE}=\frac{\mathrm{NIR}-\mathrm{RedEdge}}{\mathrm{NIR}+\mathrm{RedEdge}}$
WDRVI (wide dynamic range vegetation index) [40,41]	$\mathrm{WDRVI}=\frac{0.1\mathrm{NIR}-\mathrm{Red}}{0.1\mathrm{NIR}+\mathrm{Red}}$
MSR (modified simple ratio index) [42]	$\mathrm{MSR}=\frac{\mathrm{NIR}/\mathrm{Red}-1}{\sqrt{\mathrm{NIR}/\mathrm{Red}}+1}$
GNDVI (green normalized differential vegetation index) [43]	$\mathrm{GNDVI}=\frac{\mathrm{NIR}-\mathrm{Green}}{\mathrm{NIR}+\mathrm{Green}}$
RVI (ratio vegetation index) [44]	$\mathrm{RVI}=\frac{\mathrm{NIR}}{\mathrm{Red}}$
LCI (leaf surface chlorophyll index) [45]	$\mathrm{LCI}=\frac{\;\mathrm{NIR}-\mathrm{RedEdge}}{\;\mathrm{NIR}+\mathrm{Red}}$
OSAVI (optimize soil regulation vegetation index) [46]	$\mathrm{OSAVI}=\frac{\;\mathrm{NIR}-\mathrm{Red}}{\;\mathrm{NIR}+\mathrm{Red}+0.16}$
SAVI (soil modified vegetation index) [47]	$\mathrm{SAVI}=1.5\frac{\mathrm{NIR}-\mathrm{Red}}{\mathrm{NIR}+\mathrm{Red}+0.5}$
EVI (enhanced vegetation index) [48]	$\mathrm{EVI}=2.5\frac{\;\mathrm{NIR}-\mathrm{Red}}{\;\mathrm{NIR}+6\mathrm{Red}-7.5\mathrm{Blue}+1}$

**Table 4:** Best estimation model for canopy coverage.

Vegetation Index	Optimal Estimation Model	*R* ^2^	RMSE
NDVI	*y* = 90.589 + 40.618ln*x*	0.835	6.135
NDRE	*y* = 125.46 + 40.12ln*x*	0.802	6.714
LCI	*y* = 108.4 + 35.22ln*x*	0.83	6.217
OSAVI	*y* = 90.13 + 39.55ln*x*	0.855	5.753
EVI	*y* = 70.78*x* + 31.06	0.853	5.796
PLSR	CC = 32.84NDVI + 26.39OSAVI + 31.824EVI + 16.563	0.934	1.85

**Table 5:** Parameter correction initial value and value range.

Model Parameter	Value	Scope
*CC*_ini_	60	57–63
*den*	5000	4750–5250
*mcc*	70	66–74
*wp*	12	11.4–12.6
*hi*	5	4.75–5.25
*kcb*	0.8	0.76–0.84
*Tmg*	7	6.65–7.35
*Tupper*	30	28.5–31.5
*Tbase*	7	6.65–7.35

## Data Availability

According to relevant agreements, the data used in this study cannot be directly provided, and some relevant data can be queried from the China Meteorological Network (https://data.cma.cn/ accessed on 22 May 2022).
